# Blistering disorders and their impact on women and their families in the *International Journal of Women’s Dermatology*: Honoring the contributions of Professor Dedee Murrell

**DOI:** 10.1097/JW9.0000000000000001

**Published:** 2022-03-22

**Authors:** Maryam Daneshpazhooh, Akaterina Patsatsi, Snejina Vassileva, Jenny E. Murase

**Affiliations:** Autoimmune Bullous Diseases Research Center, Department of Dermatology, Tehran University of Medical Sciences, Tehran, Iran; 2nd Department of Dermatology, Aristotle University School of Medicine, Papageorgiou General Hospital, Thessaloniki, Greece; Department of Dermatology and Venerology, Medical University of Sofia, Sofia, Bulgaria; Department of Dermatology, University of California, San Francisco, California, Department of Dermatology, Palo Alto Foundation Medical Group, Mountain View, California

Autoimmune bullous diseases (AIBDs) are among the most fascinating areas of clinical dermatology. Despite recent advances, research is ongoing into the understanding of their pathogenesis, the detection of target antigens, and the development of nonsteroidal treatments, and only scarce data are available concerning the impact of gender on these uncommon but often life-threatening disorders. Due to their chronicity, recurrent nature, and, most importantly, the adverse effects of existing therapeutic strategies, AIBDs severely impact the quality of life of affected patients and their families. Since its inception, the *International Journal of Women’s Dermatology* has actively recruited articles from experts around the globe to discuss how blistering diseases influence women and their families, and how these diseases impact their personal and professional lives. We are highlighting these articles in a Special Collection on Blistering Disease to honor the Founding Editor and current co-Editor in Chief of *International Journal of Women’s Dermatology* Professor Dedee F. Murrell, one of the world’s leading experts on blistering diseases and an exemplary representative in the field of women’s dermatology.

Professor Murrell has made a significant contribution to AIBDs as well as the field of blistering disease at large. She is renowned for the definition of terms, the development of outcome measures, and the validation of scoring indexes for patients with both inherited and autoimmune blistering diseases. The use of these instruments is imperative for evaluating disease severity, treatment response, and quality of life. Every year, she hosts the International Bullous Diseases Group at the American Academy of Dermatology, and she has organized consensus and guideline meetings that have advanced the field for the European Academy of Dermatology and Venereology, the European Society for Dermatological Research, the Society for Investigative Dermatology, and the Japanese Society for Investigative Dermatology. Furthermore, she has pioneered the design of studies for new treatments in orphan diseases, such as Bruton tyrosine kinase inhibition in pemphigus. Her work has been recognized by the International Pemphigus & Pemphigoid Foundation. Dedee F. Murrell has been a Professor in the Faculty of Medicine at the University of New South Wales, in Sydney, Australia, since 2008. She was the first female full professor of dermatology in Australia and developed Australia’s first clinical trial center in dermatology in 1996. In addition, she edited the first textbook entirely devoted to blistering diseases.^1^ During her tenure at her department, Professor Murrell mentored more than 200 overseas fellows and medical students as well as more than 100 individuals from North America, South America, Asia, and Europe, generously giving her time during mentorships ranging from 1 month to 2 years.

Professor Murrell invited international colleagues who specialize in blistering diseases to develop articles for the journal. She has helped to enhance the role of women in dermatology with her sympathetic, enthusiastic, and welcoming attitude, as well as her passion and love for patients with blistering diseases. Professor Murrell has always been an inspiration to us as a devoted mother and wife, an excellent clinician, a great mentor, a dedicated researcher, and a passionate traveler. Both the *International Journal of Women’s Dermatology* and the Women’s Dermatologic Society owe a debt of gratitude to Professor Murrell for her 8 years of service to the organization in the role of Founding Editor and co-Editor in Chief, for the creative energy that she invested in developing articles for the journal,^2–16^ and for her solicitation of articles from world-renowned experts^17–30^ that have improved the lives of women and their families throughout the world with blistering diseases.

## Conflicts of interest

None.

## Acknowledgments

We thank Jennifer Ehrhardt for her assistance in identifying the articles involving blistering disease since the inception of the journal for this article. Ms Ehrhardt has been a dedicated and hardworking Managing Editor of the *International Journal of Women’s Dermatology*, and we are indebted to her for diligence, enthusiasm, determination, and commitment to our vision of improving the dermatologic health of women and their families throughout the world.

## Funding

None.

## Study approval

The author(s) confirm that any aspect of the work covered in this manuscript that has involved human patients has been conducted with the ethical approval of all relevant bodies.
